# CIDP With and Without Monoclonal Gammopathy of Undetermined Significance (MGUS): Comparison of Clinical Phenotype, Diagnostic Features, and Treatment Response

**DOI:** 10.1111/jns.70116

**Published:** 2026-03-12

**Authors:** R. van Veen, A. E. Baars, I. N. van Doorn, M. Michael, S. R. M. Bus, M. C. Broers, W. L. van der Pol, P. A. Van Doorn, J. Drenthen, C. Verhamme, J. M. I. Vos, I. N. van Schaik, H. S. Goedee, L. Wieske, B. C. Jacobs, F. Eftimov, for the ICOS Consortium

**Affiliations:** ^1^ Department of Neurology Amsterdam UMC Location University of Amsterdam Amsterdam the Netherlands; ^2^ Amsterdam Neuroscience Neuroinfection and Inflammation Amsterdam the Netherlands; ^3^ Department of Psychiatry OLVG Hospital Amsterdam the Netherlands; ^4^ Department of Neurology, Erasmus MC University Medical Center Rotterdam the Netherlands; ^5^ Department of Neurology, Brain Center Rudolf Magnus University Medical Center Utrecht the Netherlands; ^6^ Department of Haematology, Cancer Center Amsterdam Amsterdam UMC Location University of Amsterdam Amsterdam the Netherlands; ^7^ Sanquin Blood Supply Foundation Amsterdam the Netherlands; ^8^ Department of Clinical Neurophysiology St. Antonius Hospital Nieuwegein the Netherlands; ^9^ Department of Immunology, Erasmus MC University Medical Center Rotterdam the Netherlands

**Keywords:** chronic inflammatory demyelinating polyneuropathy (CIDP), clinical phenotype, monoclonal gammopathy of undetermined significance (MGUS), nerve conduction studies, paraproteinemia, treatment response

## Abstract

**Background and Aims:**

Monoclonal gammopathy of undetermined significance (MGUS) occurs in some patients with chronic inflammatory demyelinating polyneuropathy (CIDP), but its impact on clinical phenotype and treatment response remains unclear. We assessed the prevalence of paraproteinemia in CIDP and compared disease features between CIDP patients with and without MGUS.

**Methods:**

We used data from the International CIDP Outcome Study (ICOS), a prospective cohort study. We compared the prevalence and causes of paraproteinemia in CIDP to matched disease controls (axonal polyneuropathy or motor neuron disease) and compared disease features and treatment responses between CIDP patients with and without MGUS. Treatment response, defined as a ≥ 1‐point improvement on the modified Rankin scale, was retrospectively assessed.

**Results:**

IgG paraproteinemia was more common in CIDP than in controls (9%, 17/193 vs. 3%, 6/192; *p* = 0.03). IgM and IgA paraprotein prevalences did not differ. One CIDP patient had Waldenström macroglobulinemia; others had MGUS. Patients with IgG MGUS less often had an acute clinical presentation (6% vs. 33%; *p* = 0.02), more often had sensory deficits (94% vs. 67%; *p* = 0.02), and prolonged distal CMAP duration (64% vs. 31%; *p* = 0.02), compared to patients without MGUS. First‐line treatment response rates were comparable (80% [IgG MGUS] vs. 67% [no MGUS]; *p* = 0.39).

**Interpretation:**

IgG MGUS is more prevalent in CIDP than in controls. Presence of IgG MGUS is weakly associated with some CIDP disease features, but not treatment response. These findings indicate that, although IgG MGUS is associated with CIDP, the presence of IgG MGUS does not constitute a distinct subgroup with unique clinical features or treatment implications.

## 1 Introduction

Chronic inflammatory demyelinating polyneuropathy (CIDP) is a rare, clinically heterogeneous disease with an incompletely understood immunopathogenesis [1]. An important step in the diagnostic work‐up of CIDP is to assess the presence of paraproteinemia, as it may point toward an alternative diagnosis. An IgM paraprotein, when found alongside anti‐myelin associated glycoprotein (MAG) antibodies and a distal clinical phenotype, may suggest an anti‐MAG polyneuropathy. In contrast, IgA or IgG paraproteins are associated with conditions such as POEMS syndrome (polyneuropathy organomegaly, endocrinopathy, monoclonal protein, and skin changes) and amyloidosis [2].

Paraproteins are produced by monoclonal plasma cells, plasmablasts or B‐cells, mostly in the bone marrow (BM). Clonal BM infiltration exceeding 10% indicates hematological malignancies, such as multiple myeloma or Waldenström macroglobulinemia [3]. However, most paraproteins are detected in the context of monoclonal gammopathy of undetermined significance (MGUS), a premalignant condition defined by < 10% BM infiltration. The majority of MGUS patients however never progress to malignancy [4, 5]. MGUS is relatively common in the general population, with a prevalence of 3% in individuals over 50 years, and is usually of the IgG isotype [6]. This prevalence increases with age and is higher in males [6].

MGUS is generally thought to be unrelated to CIDP but this is questionable, as the prevalence of IgM, IgG, and IgA paraproteinemia is higher in patients with CIDP compared to the general population [7, 8]. In addition, it remains unclear whether the presence of MGUS in CIDP patients indicates a pathophysiologically distinct subgroup with different clinical characteristics, diagnostic findings, or treatment responses compared to CIDP patients without MGUS.

Therefore, the aims of this study are (1) to determine the prevalence of paraproteinemia in a large cohort of patients with CIDP and compare these findings to matched neurological controls and (2) to assess differences in clinical characteristics, diagnostic features, and treatment responses in patients with CIDP with and without MGUS.

## 2 Materials and Methods

### 2.1 Patients

We used data from the International CIDP Outcome Study (ICOS) [9], a prospective observational cohort study in patients with CIDP that included patients in three large Dutch tertiary neuromuscular centers: the Erasmus University Medical Center (EMC) in Rotterdam, the Amsterdam University Medical Centers (Amsterdam UMC), location AMC, and the Utrecht Medical University Center (UMCU). The ICOS research protocol has been published previously [9]. In short, inclusion criteria allowed both newly and previously diagnosed patients with definite, probable, or possible CIDP based on the 2010 European Federation of Neurological Societies/Peripheral Nerve Society criteria (2010 criteria) [10]. Patients who did not meet the electrodiagnostic criteria but fulfilled the clinical criteria for CIDP and at least two supportive criteria were also included [11]. Patients with other identifiable causes for demyelinating neuropathy were excluded, including those whose diagnosis was revised during follow‐up, thereby excluding CIDP mimics such as POEMS syndrome from the cohort. Because the EFNS/PNS 2010 diagnostic criteria were used [10], patients with paranodal antibodies were not excluded from the ICOS cohort. The study was approved by the Medical Ethics Committees of the participating centers, and written informed consent was obtained from all participating ICOS patients. In this study, we included all patients with CIDP who were screened for paraproteins as part of their diagnostic work‐up and were enrolled before the start of the current data analyses (November 2015 until January 2020). Patients who tested positive for anti‐MAG antibodies were excluded, regardless of antibody titer.

The neurological controls consisted of patients who underwent an extensive nerve conduction study (NCS) protocol at the Amsterdam UMC for the evaluation of suspected demyelinating neuropathy, from whom retrospective data were used. No informed consent was required for inclusion of these patients, as judged by the Medical Ethics Committee, but all patients received an objection letter. Patients who objected to the use of their data were excluded from the study. We included controls with a final diagnosis of an axonal polyneuropathy or motor neuron disease (based on clinical features, NCS findings, cerebrospinal fluid [CSF], nerve ultrasound, MRI, nerve biopsy and an objective treatment response) if screening for paraproteins was performed and if diagnostic work‐up was performed before January 2020. We included these controls because neither axonal polyneuropathy nor motor neuron disease is associated with MGUS [8]. We selected the controls that best matched the ICOS patients in terms of age and sex, as paraproteinemia prevalence rates vary based on these characteristics [6].

### 2.2 Paraprotein Screening and Hematological Diagnosis

We extracted paraprotein screening results, age at screening and the methods of screening (this varied based on location and period of screening), including isotypes and paraprotein concentrations for patients with CIDP and neurological controls. Screening panels for patients with polyneuropathy did not include routine assessments of urine or serum free light chains. BM examination results and hematology reports around the time of screening were obtained, and for patients with CIDP, also at follow‐up. The treating physicians (internal medicine specialists or hematologists) indicated the need for additional hematological examinations, as BM examination can be deferred in cases of a low risk of a malignancy, depending on the concentration of the paraprotein, among other factors [12].

For this study, patients were classified according to the International Myeloma Working Group criteria [3], based on paraprotein concentration and, when available, BM examination results. We classified patients as “confirmed MGUS” if the paraprotein concentration was < 30 g/L and BM infiltration was < 10%. If the paraprotein concentration was < 30 g/L, but no BM examination was performed or available, we classified patients as “unconfirmed MGUS.” If the concentration of the paraprotein or BM infiltration exceeded these cut‐offs, patients were diagnosed based on the diagnostic criteria of B‐cell or plasma cell malignancies.

### 2.3 Clinical Evaluation

Data regarding the demographics, comorbidity, symptoms at onset, examination at diagnosis, clinical phenotype and treatment details were collected according to the predefined ICOS research protocol. Symptoms at onset were obtained by taking a patient history. Acute clinical presentation was scored by the investigators based on clinical notes and their experience with different CIDP time courses. From the examination at diagnosis, we recorded the presence of proximal weakness, distal weakness, sensory dysfunction, ataxia, tremor, autonomic dysfunction and pain (rated as: yes, no, or unknown), either retrospectively from medical correspondence for previously diagnosed patients at ICOS entry, or carried out at ICOS entry for newly diagnosed patients.

From newly diagnosed patients with CIDP at ICOS entry, we also collected the following outcome measures: the Medical Research Council sum score (MRC‐SS, ranging from 0 [severe weakness] to 60 points [normal strength]) [13], grip strength of both the weakest and strongest hand (to avoid over‐ or underestimation of weakness in asymmetric variants) measured using a Martin‐Vigorimeter (ranging from 0 [severe weakness] to 160 [no weakness] kPa, best out of three measurements) [14, 15], the modified Inflammatory Neuropathy Cause and Treatment Sensory Score (INCAT‐SS, ranging from 0 [no sensory deficits] to 33 points [severe sensory deficits]) [16], and the inflammatory Rasch‐built Overall Disability Scale (I‐RODS, ranging from 0 [severe disability] to 100 centiles [no disability]) [17]. We made separate MRC‐SS for distal weakness (including wrist extension and ankle flexion, ranging from 0 to 20 points) and proximal weakness (including shoulder abduction, elbow flexion, hip flexion, and knee extension, ranging from 0 to 40 points), to test for differences in the distribution of weakness.

Both newly and previously diagnosed CIDP patients at ICOS entry were retrospectively assessed using the Modified Rankin scale (mRS) before and after starting their first treatment for CIDP.

### 2.4 Diagnostic Investigations

We obtained results of serum antibodies (anti‐GM1, anti‐GQ1b, anti‐GD1b [tested using ELISA], anti‐CASPR1, anti‐CNTN1 and anti‐NF155 [tested using cell‐based assays (CBA)]; rated as: present, not present or not tested), that were obtained as part of the diagnostic process (at diagnosis or at follow‐up) at the discretion of the treating physician based on clinical suspicion. In the context of a previous study, we collected serum samples at ICOS inclusion of patients included in the EMC and Amsterdam UMC. These were tested for anti‐CASPR1, anti‐CNTN1 and anti‐NF155 using CBA (rated as: present, not present or not tested), including IgG subclasses when paranodal antibodies were found (for details, see reference [18]). We also collected CSF examinations, MRI scans, nerve ultrasounds and nerve biopsies (each rated as: supportive of CIDP, not supportive of CIDP or not tested), that were obtained as part of the diagnostic process at the discretion of the treating physician. NCS were conducted at time of diagnosis in all patients included in the EMC and Amsterdam UMC. We did not have access to detailed full NCS reports of patients included in the UMCU. The extensiveness of the NCS was based on the local protocols at the time of the execution of the NCS and the individual patient characteristics. NCS were reassessed by clinical neurophysiologists experienced in CIDP, according to the electrodiagnostic 2010 criteria and 2021 EAN/PNS criteria for CIDP (2021 criteria) [10, 19]. When available, bilateral assessments for features supportive of demyelination were carried out using the diagnostic motor nerve conduction criteria (either meeting or not meeting criteria), for segments of the median, ulnar, radial, musculocutaneous, peroneal and tibial nerves, without severely reduced (1 mV) compound muscle action potential (CMAP) amplitudes. The median, ulnar, radial and sural nerve were bilaterally assessed using the sensory conduction criteria (either meeting or not meeting the criteria), when available. We classified each patient into “definite CIDP,” “probable CIDP,” “possible CIDP,” and “no CIDP” based on the electrodiagnostic and diagnostic 2010 criteria [10]. Using the 2021 motor nerve conduction criteria, we classified the NCS of patients into “strongly supportive of demyelination,” “weakly supportive of demyelination,” or “not supportive of demyelination.” Also, we classified patients according to the electrodiagnostic 2021 criteria [19], into “CIDP,” “possible CIDP” or “no CIDP,” by taking into account the clinical variants and their corresponding electrodiagnostic criteria, including the motor conduction criteria and sensory conduction criteria. Next, we classified patients according to the diagnostic 2021 criteria, into either “CIDP,” “possible CIDP” or “no CIDP,” using also the supportive criteria, consisting of CSF, nerve ultrasound, MRI, nerve biopsy and/or an objective treatment response (see paragraph below) [10, 19].

### 2.5 Treatment Response

From patients with MGUS, we extracted treatment details from all received treatments, including the type of treatment and the start and stop dates of each treatment. A period of remission was defined as a period of ≥ 6 months without treatment.

For evaluation of an objective treatment response used as supportive criterion for the diagnostic 2021 criteria [19], we used prospective data when available. We defined a treatment response as an improvement greater than the described cut‐off on at least one impairment scale (MRS‐SS ≥ 2 points, INCAT‐SS ≥ 2 points and/or grip strength ≥ 8 kPA) and on one disability scale (I‐RODS ≥ 4 centile points and/or the Inflammatory Neuropathy Cause and Treatment disability score ≥ 1 point) [19], within 6 months after starting treatment. If prospective data was not available, we used retrospective data and defined an objective treatment response as an improvement of ≥ 1 point on the mRS, within 6 months after starting treatment.

We compared treatment response to the first treatment between patients with and without MGUS, using the retrospectively assessed mRS, as most patients were on maintenance treatment at baseline. A response was defined as described above.

### 2.6 Analysis

First, we tested whether the prevalence of IgG, IgM, IgA, or biclonal paraproteinemia, as well as elevated free light chains, differed between patients with CIDP and neurological controls. Dubious paraprotein results were regarded as negative for this comparison.

For patients with paraproteinemia, the paraprotein concentration was categorized as “not quantifiable,” “< 5,” “5–10,” and “10–15” and “>15 g/L”.

Second, for patients with MGUS, we grouped patients with CIDP based on their heavy chain isotype and tested for differences in clinical characteristics, results of diagnostic investigations and treatment response between patients with MGUS (heavy chain isotypes separately) and patients without. Patients with biclonal or dubious paraprotein results, or with paraproteinemia related to a B‐cell or plasma cell malignancy around the time of screening, were excluded from the analyses of clinical characteristics, results of diagnostic investigations and treatment response.

Since the start of the ICOS study, new diagnostic criteria for CIDP have been implemented. Therefore, we conducted sensitivity analyses selecting CIDP patients based on the 2021 diagnostic criteria [19]. In this dataset, we selected patients of whom we had access to the NCS, and excluded patients with paranodal antibodies and patients who were classified as “no CIDP” using the diagnostic 2021 criteria (see section above: “diagnostic investigations”) [19].

Numeric variables are presented as means with standard deviations or median values with interquartile ranges, as appropriate. Categorical variables are presented as percentages, and for paraprotein prevalence rates also 95% confidence intervals. We tested for group differences in (semi) continuous variables using a *t*‐test (for normally distributed data) or the Mann–Whitney *U* test (for nonnormally distributed data or small sample sizes). For binary variables, we used the Fisher”s exact test to test for group differences, because of our small sample size. When variables had more than two categories, we applied the extended version of Fisher's exact test (Fisher–Freeman–Halton) and, if the overall test was significant, performed pairwise comparisons to identify specific group differences.

We did not correct for multiple comparisons due to the explorative nature of this study. A *p*‐value of < 0.05 was considered significant.

Analyses were performed in R (version 4.0.2.) [20], a software environment for statistical computing and graphics.

## 3 Results

### 3.1 Paraprotein Screening and Hematological Diagnosis

Paraprotein screening was conducted in 91% (194/213) of patients with CIDP during their diagnostic work‐up and was included in this study. One patient had IgM paraproteinemia with anti‐MAG and was excluded from this study. For analyses of clinical characteristics, diagnostic investigations, and treatment responses in CIDP patients, we excluded patients with B‐cell or plasma cell malignancies (*n* = 1), elevated free light chains (*n* = 1), and biclonal (*n* = 1) or dubious paraprotein results (*n* = 2, Table 1), resulting in a total of 17 patients with IgG MGUS, 6 patients with IgM MGUS, and 165 patients without MGUS.

**TABLE 1:** Paraprotein screening of patients with CIDP and neurological controls.

	CIDP (*n* = 193)	Neurological controls (*n* = 192)^a^	*p*
Age at screening	57 years (15)	63 years (13)	**0.01**
Gender (males)	69% (134/193)	64% (122/192)	0.23
Method of screening^b^			
Serum IFX and EF	51% (88/172)	69% (127/184)	
Pentascreening	1% (1/172)	1% (1/184)	**< 0.01** ^c^
Capillary EF	10% (17/172)	1% (1/184)	
EF or protein spectrum	38% (66/172)	30% (56/184)	
Prevalence			
IgG	9% (5%–13%; 17/193)^d^	3% (1%–6%; 6/192)	**0.03**
LC type	53% *κ*; 47% *λ*	50% *κ*; 50% *λ*	
IgM	4% (1%–6%; 7/193)	2% (0%–3%; 3/192)^g^	0.33
LC type	71% *κ*; 29% *λ*	67% *κ*; 33% *λ*	
IgA	0% (0%–0%; 0/193)	1% (0%–2%; 2/192)	0.25
LC type	—	100% *κ*	
Biclonal	1% (0%–2%; 1/193)^e^	1% (0%–2%; 2/192)^h^	0.62
IgG λ and IgM λ	100% (1/1)	0% (0/2)	
IgG λ and IgA λ	0% (0/1)	50% (1/2)	
IgM λ and IgM κ	0% (0/1)	50% (1/2)	
Free light chains	1% (0%–2%; 1/193)^f^	0% (0%–0%; 0/192)^i^	1.00
Paraprotein concentration^j^			
Not quantifiable	52% (13/25)	0% (0/12)	0.73^k^
< 5 g/L	44% (11/25)	75% (9/12)	
5–10 g/L	4% (1/25)	8% (1/12)	
10–15 g/L	0% (0/25)	17% (2/12)	
> 15 g/L	0% (0/25)	0% (0/12)	
Hematological diagnosis^l^			
MGUS	96% (25/26)	100% (13/13)	1.00
Confirmed	36% (9/25)	54% (7/13)	
Unconfirmed	64% (16/25)	46% (6/13)	
B‐cell or plasma cell malignancy	4% (1/26)^m^	0% (0/13)	
Malignant transformation during FU	4% (1/25)^n^	NA	

*Note:* Numeric data are presented as means and standard deviations (for normal distributed data; mean [SD]) or medians and interquartile range (for nonnormally distributed data; median [interquartile range]). Categorical variables are presented as percentages (with 95% confidence intervals for paraprotein prevalence rates) and count/total (percentage [95% CI; count/total] or percentage [count/total]). Statistically significant results (*p*‐value < 0.05) are presented in bold.

Abbreviations: *κ*: kappa; *λ*: lamba; CIDP: chronic inflammatory demyelinating polyneuropathy; EF: electrophoresis; FU: follow up; IFX: serum immunofixation; LC: light chain.

^a^ Axonal neuropathy: 38%; motor neuron disease: 62%.

^b^ Method of screening was unknown in 21 ICOS patients and 8 controls.

^c^ With post hoc pairwise Fisher's test: significant difference in “serum IFX and EF” and “capillary EF.”

^d^ In addition: two patients had a dubious IgG kappa paraprotein (g/L paraprotein not quantifiable and total IgG concentration within normal range).

^e^ One patient with both IgM lambda (1.9 g/L) and IgG lambda (2.9 g/L) paraproteins.

^f^ One patient with elevated kappa light chain (42.4 g/L).

^g^ One patient with an IgM lambda paraprotein had also dubious IgG lambda (g/L not quantifiable) paraprotein. In addition: one patient had a dubious IgM kappa paraprotein (g/L not quantifiable) and one patient had a dubious IgM lambda paraprotein (g/L not quantifiable).

^h^ One patient with both IgG lambda (2.1 g/L) and IgA lambda (g/L not quantifiable) paraproteins and one patient with IgM lambda (0.2 g/L) and IgM kappa (0.4 g/L) paraproteins.

^i^ In addition: one patient had dubious elevated free light chain, of which the type light chain was unable to determine (kappa/lambda ratio: 0.49).

^j^ Only for patients with paraproteins. One patient with elevated kappa light chain had a concentration of 42.4 g/L. For patients with biclonal paraproteinemia, we used the largest quantity of the two paraproteins for categorization. Concentration was unknown for one control patient.

^k^ Comparison between categories: “< 5 g/L,” “5–10 g/L,” “10–15 g/L,” and “> 15 g/L.”

^l^ Hematological diagnosis of paraprotein or elevated free light chains, at around screening, in case a paraprotein or elevated free light chains was found. When paraprotein concentrations were < 30 g/L and bone marrow infiltration was < 10%, patients were classified as confirmed MGUS. When paraprotein concentrations were < 30 g/L, but no bone marrow examination was executed or available, we classified patients as unconfirmed MGUS.

^m^ One patient with CIDP and IgM paraproteinemia at screening was diagnosed with Waldenström macroglobulinemia within 2 months after screening.

^n^ One patient diagnosed with multiple myeloma was diagnosed 3 years after screening.

We selected and included 192 out of 247 neurological controls, based on matching on gender and age, of whom 73 were diagnosed with axonal polyneuropathy and 119 with motor neuron disease.

The results of paraprotein screening of patients with CIDP and neurological controls are summarized in Table 1. In the CIDP group, 17 patients had IgG paraproteinemia, 7 had IgM paraproteinemia, 1 had biclonal paraproteinemia (IgM and IgG), 1 had elevated free light chains, and none had IgA paraproteinemia. The prevalence of IgG paraproteinemia was significantly higher in patients with CIDP compared to neurological controls: 9% (95% CI: 5%–13%; 17/193) vs. 3% (95% CI: 1%–6%; 6/192; *p* = 0.03). The prevalence of IgM paraproteinemia did not differ significantly between patients with CIDP and neurological controls (4%, 95% CI: 1%–6%, 7/193 [CIDP] vs. 2%, 95% CI: 0%–3%, 3/192 [controls]; *p* = 0.33), nor did the prevalence of IgA paraproteinemia (0%, 95% CI: 0%–0%, 0/193 [CIDP] vs. 1%, 95% CI: 0%–2%, 2/192 [controls]; *p* = 0.25).

One patient with CIDP and IgM paraproteinemia at screening was diagnosed with Waldenström macroglobulinemia within 2 months after screening and was excluded from further analyses. Other patients with CIDP had MGUS, which was confirmed in 36% by BM examination. During follow‐up, in one CIDP patient, IgG MGUS transformed to multiple myeloma 3 years after paraprotein screening. Details of all patients with paraproteinemia can be found in Table S1.

### 3.2 Clinical Characteristics, Diagnostic Investigations and Treatment Response in Patients With CIDP and IgG MGUS

Table 2 summarizes the clinical characteristics of patients with CIDP with IgG MGUS and CIDP without MGUS. A typical clinical phenotype of CIDP was observed in 82% (14/17) of IgG MGUS patients and 73% (121/165) of those without MGUS (*p* = 0.62 when comparing all clinical phenotype categories). An acute clinical presentation was more often found in patients without MGUS (33%, 54/165) compared to those with IgG MGUS (6%, 1/17; *p* = 0.02). Sensory deficits at onset were more common in IgG MGUS patients, while other symptoms were comparable compared to patients without MGUS.

**TABLE 2:** Clinical characteristics and treatment response of patients with CIDP with IgG MGUS and without MGUS.

	CIDP with IgG MGUS, *n* = 17	CIDP without MGUS, *n* = 165	*p*
Gender (males)	71% (12/17)	68% (113/165)	1.00
Age at CIDP diagnosis	63 years (9)	61 years (14)	0.27
Other AI disease	0% (0/17)	13% (22/164)	0.23
Onset to ICOS entry	3 years (1–10)	2 years (1–7)^a^	0.18
Diagnosis to ICOS entry	2 years (0–9)	0 years (0–3)^b^	0.20
Phenotype			
Typical	82% (14/17)	73% (121/165)	
Variants			
(Multi) focal	6% (1/17)	14% (23/165)	
Distal	6% (1/17)	3% (4/165)	0.62
(Predominantly) motor	6% (1/17)	6% (10/165)	
(Predominantly) sensory	0% (0/17)	4% (6/165)	
Disease onset			
Acute clinical presentation^c^	6% (1/17)	33% (54/165)	**0.02**
Onset to diagnosis	1 year (1–1)	1 year (0–2)	0.26
Weakness	35% (6/17)	57% (94/165)	0.12
Sensory deficits	94% (16/17)	67% (110/165)	**0.02**
Cranial nerve deficits	0% (0/17)	5% (9/165)	1.00
Gait disturbances	18% (3/17)	18% (30/165)	1.00
Examination at diagnosis			
Weakness	82% (14/17)	97% (149/154)^e^	0.14
Proximal	44% (7/16)^d^	67% (107/160)^b^	0.10
Distal	81% (13/16)^a^	91% (145/160)^b^	0.21
Sensory dysfunction	100% (17/17)	91% (145/159)^f^	0.37
Ataxia	12% (2/17)	5% (9/165)	0.27
Tremor	18% (3/17)	18% (29/165)	1.00
Autonomic dysfunction	0% (0/17)	6% (10/165)	0.60
Pain	18% (3/17)	27% (42/153)^g^	0.56
Newly diagnosed patients at entry^h^	*n* = 5	*n* = 52	
Typical phenotype	60% (3/5)^o^	75% (39/52)^p^	0.60
MRC‐SS (points)^i^	56 (56–60)	54 (50–57)	0.15
Proximal^j^	38 (38–40)	36 (34–40)	0.27
Distal^k^	18 (18–20)	17 (16–18)	0.21
Grip strength (kPa)^l^			
Weakest hand	71 (50–78)	45 (30–70)	0.19
Strongest hand	86 (60–88)	50 (41–78)	0.21
INCAT‐SS (points)^m^	7 (2–7)	6 (4–9)	0.35
I‐RODS (centile)^n^	71 (57–83)	62 (47–71)	0.39
Treatment response			
Treatment received	94% (13/17)	96% (158/165)	
Responder^q^	80% (12/15)	67% (94/140)	0.39
IVIg monotherapy	80% (8/10)	69% (63/91)^s^	
Corticosteriods monotherapy	100% (2/2)	65% (13/20)	
IVIg and corticosteroids	100% (3/3)^r^	61% (17/28)^p^	
Other	(0/0)	100% (2/2)^t^	

*Note:* Numeric data are presented as means and standard deviations (for normal distributed data; mean [SD]) or medians and interquartile range (for nonnormally distributed data; median [interquartile range]). Categorical variables are presented as percentages and count/total (percentage [count/total]).

Abbreviations: AI: autoimmune; CIDP: chronic inflammatory demyelinating polyneuropathy; INCAT‐SS: modified inflammatory neuropathy cause and treatment sensory score; I‐RODS: the inflammatory Rasch‐Overall Disability Scale; IVIg: intravenous immunoglobulins; kPa: kilopascal; MRC‐SS: Medical Research Council sum score. Statistically significant results (*p*‐value < 0.05) are presented in bold.

^a^ Year of onset unknown in 11 patients.

^b^ Year of diagnosis unknown in one patient.

^c^ Acute clinical presentation was scored by the investigators based on clinical notes and their experience with different CIDP time courses.

^d^ Unknown in 1 patient.

^e^ Unknown in 11 patients.

^f^ Unknown in six patients.

^g^ Unknown in 12 patients.

^h^ Newly diagnosed patients at ICOS entry (IgG MGUS: *n* = 5; no MGUS: *n* = 52).

^i^ Ranging from 0 (severe weakness) to 60 points (normal strength).

^j^ MRC sum score of arm abduction, elbow flexion, hip flexion and knee extension, bilaterally (ranging from 0 [severe weakness] to 40 points [normal strength]).

^k^ MRC sum score of wrist extension and ankle dorsiflexion, bilaterally (ranging from 0 [severe weakness] to 20 points [normal strength]).

^l^ Ranging from 0 (severe weakness) to 160 kPa (no weakness), best out of three measurements.

^m^ Ranging from 0 (no sensory deficits) to 33 points (severe sensory deficits).

^n^ Ranging from 0 (severe disability) to 100 centiles (no disability).

^o^ Atypical variants: 1/5 distal, 1/5 pure motor.

^p^ Atypical variants: 9/52 (multi)focal; 1/52 distal, 2/52 pure motor, 1/52 pure sensory.

^q^ A responder was defined as a patient that improved ≥ 1 point on the mRS upon the first treatment. Based on the available data we were able to classify treatment response in 89% of patients using the mRS (in 15/16 patients with IgG MGUS and 140/158 of patients without MGUS).

^r^ One patient participated in the OPTIC trial.

^s^ Eight patients participated in the OPTIC trial.

^t^ One patient was treated with pulsed corticosteroids and plasmapheresis, and one patient was treated with plasmapheresis.

**FIGURE 1:**
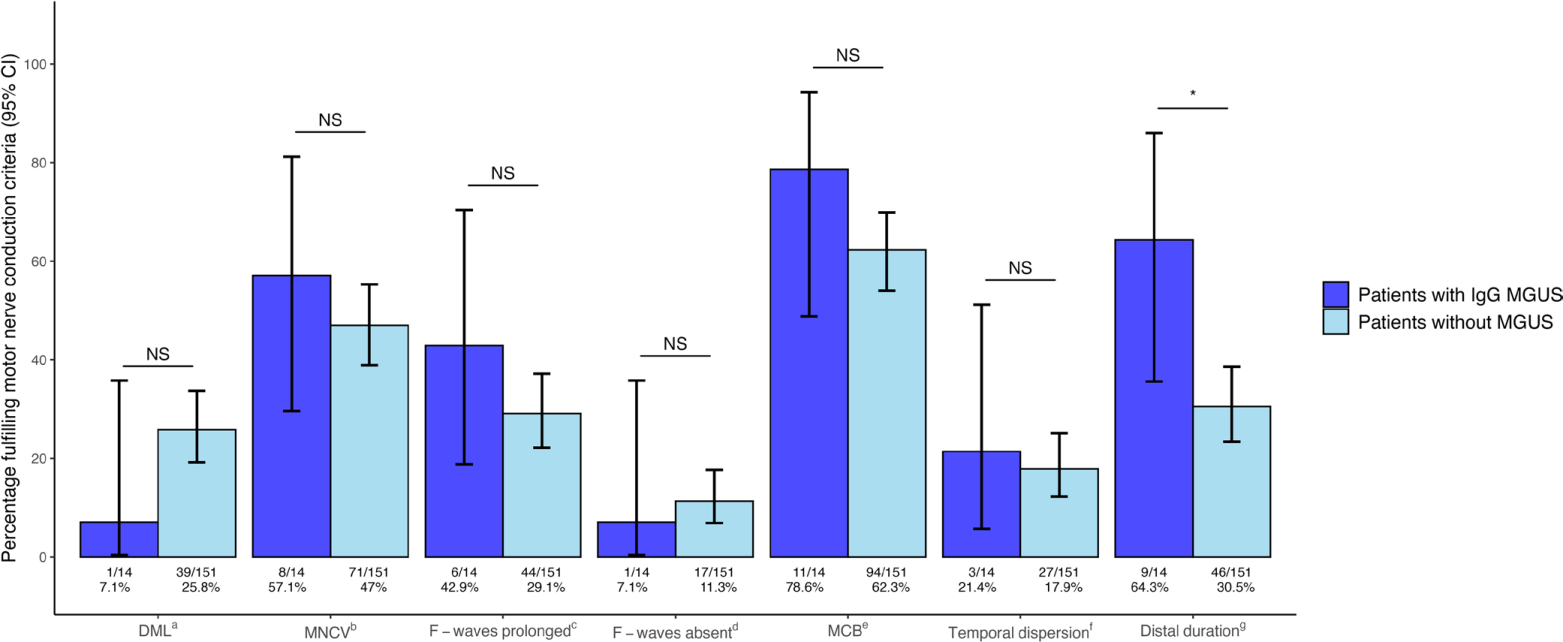
Motor nerve conduction criteria fulfillment of patients with CIDP with IgG MGUS and without MGUS. Values on the *Y*‐axis represent the percentage of patients that met the motor conduction criteria (a–g) of the 2021 EAN/PNS guideline for CIDP. The bars represent the 95% confidence intervals. Only assessed for patients included in EMC or Amsterdam UMC (MGUS IgG: *n* = 14; no MGUS: *n* = 151). *p = 0.02. ^a^Criterium 1a: motor distal latency prolongation ≥ 50% above upper limit of normal values in two nerves (excluding median neuropathy at the wrist from carpal tunnel syndrome). ^b^Criterium 1b: reduction of motor conduction velocity ≥ 30% below lower limit of normal in two nerves. ^c^Criterium 1c: prolongation of F‐wave latency ≥ 20% above upper limit of normal values in two nerves (≥ 50% if amplitude of distal negative peak CMAP < 80% of lower limit of normal). ^d^Criterium 1d: absence of F‐waves in two nerves (if these nerves have distal negative peak CMAP amplitudes ≥ 20% of lower limit of normal) + ≥ 1 other demyelinating parameter in ≥ 1 other nerve. ^e^Criterium 1e: motor conduction block: ≥ 30% reduction of the proximal relative to distal negative peak CMAP amplitude, excluding the tibial nerve, and distal negative peak CMAP amplitude ≥ 20% of lower limit of normal in two nerves; or in one nerve + ≥ 1 other demyelinating parameter except absence of F‐waves in ≥ 1 other nerve. ^f^Criterium 1f: abnormal temporal dispersion: > 30% duration increase between the proximal and distal negative peak CMAP (at least 100% in the tibial nerve) in ≥ 2 nerves. ^g^Criterium 1g: distal CMAP duration prolongation in ≥ 1 nerve + ≥ 1 other demyelinating parameter in ≥ 1 other nerve. Abbreviations: CIDP: chronic inflammatory demyelinating polyneuropathy; DML: motor distal latency prolongation; EAN/PNS: European Academy of Neurology/Peripheral Nerve Society; MCB: motor conduction block; MNCV: motor nerve conduction velocity.

Table 3 presents the results of diagnostic investigations for patients with IgG MGUS and those without MGUS. Strongly supportive motor nerve conduction criteria of demyelination, based on 2021 criteria, were met in 93% (13/14) of IgG MGUS patients and 74% (112/151) of patients without MGUS (*p* = 0.42 when comparing the categories: strongly, weakly and not supportive of demyelination). Motor nerve conduction criteria fulfillment, per criterion (based on 2021 criteria) can be found in Figure 1. The motor criterion “distal CMAP duration prolongation in at least one nerve and at least one other demyelinating parameter in at least one other nerve” was more often present in patients with IgG MGUS (64%, 9/14) than in patients without MGUS (31%, 46/151; *p* = 0.02), but no other significant differences were found. The electrodiagnostic and diagnostic classifications according to the 2010 and 2021 diagnostic criteria can be found in Table 3.

**TABLE 3:** Results of diagnostic investigations of patients with CIDP with IgG MGUS and without MGUS.

	CIDP with IgG MGUS, *n* = 17	CIDP without MGUS, *n* = 165	*p*
Serum antibodies			
Anti‐GM1^a^	17% (1/6)^d^	15% (6/41)^e^	NA^f^
Anti‐GQ1b or anti‐GD1b^b^	0% (0/2)	0% (0/26)	
Anti‐CASPR1^c^	0% (0/9)	2% (2/96)	
Anti‐CNTN1^c^	0% (0/9)	2% (2/96)	
Anti‐NF155^c^	0% (0/9)	4% (4/96)	
CIDP diagnosis supported by			
CSF examination^g^	73% (11/15)	64% (84/131)	0.58
Nerve ultrasound^h^	50% (3/6)	80% (53/66)	0.12
Nerve biopsy^i^	0% (0/1)	43% (3/7)	1.00
MRI scan^j^	0% (0/6)	28% (16/58)	0.32
NCS^k^	*n* = 14	*n* = 151	
Motor nerves tested	5 (4–9)	6 (4–12)	0.61
Classification 2010 crit.			
Electrodiagnostic crit.^l^			0.69
Definite CIDP	86% (12/14)	74% (111/151)	
Probable CIDP	0% (0/14)	3% (4/151)	
Possible CIDP	14% (2/14)	13% (19/151)	
No CIDP	0% (0/14)	11% (17/151)	
Diagnostic crit.^m^			0.57
Definite CIDP	100% (14/14)	82% (124/151)	
Probable CIDP	0% (0/14)	5% (7/151)	
Possible CIDP	0% (0/14)	2% (3/151)	
No CIDP	0% (0/14)	11% (17/151)	
Classification 2021 crit.			
Motor nerve conduction crit.^n^			0.42
Strongly supportive of dem.^o^	93% (13/14)	74% (112/151)	
Weakly supportive of dem.^p^	7% (1/14)	17% (25/151)	
Not supportive of dem.^q^	0% (0/14)	9% (14/151)	
Electrodiagnostic crit.^r^			0.32
CIDP	86% (12/14)	66% (100/151)	
Possible CIDP	14% (2/14)	18% (28/151)	
No CIDP	0% (0/14)	15% (23/151)	
Diagnostic crit.^s^			0.79
CIDP	86% (12/14)	75% (112/151)	
Possible CIDP	14% (2/14)	17% (26/151)	
No CIDP	0% (0/14)	9% (13/151)	

*Note:* Numeric data are presented as means and standard deviations (for normal distributed data; mean [SD]) or medians and interquartile range (for nonnormally distributed data; median [interquartile range]). Categorical variables are presented as percentages and count/total (percentage [count/total]).

Abbreviations: CIDP: chronic inflammatory demyelinating polyneuropathy; Crit.: criteria; CSF: cerebrospinal fluid; dem.: demyelination; MRI: magnetic resonance imaging; NCS: nerve conduction studies.

^a^ Not tested in 11 CIDP patients with IgG MGUS and 124 CIDP patients without MGUS.

^b^ Not tested in 15 CIDP patients with IgG MGUS and 139 CIDP patients without MGUS.

^c^ Not tested in 8 CIDP patients with IgG MGUS and 69 CIDP patients without MGUS; one patients had both anti‐CASPR1 and anti‐CNTN1; Paranodal antibodies were of the IgG4 subclass in all 7/7 patients.

^d^ Anti‐GM1 (IgM) weak positive, anti‐GM1 (IgG) negative, multifocal phenotype.

^e^ Phenotype: 3/6 typical, 2/6 motor variant, 1/6 multifocal variant.

^f^ Differences between groups not assessed for significance due to the small sample size.

^g^ Not tested in 2 CIDP patients with IgG MGUS and 34 CIDP patients without MGUS.

^h^ Not tested in 11 CIDP patients with IgG MGUS and 99 CIDP patients without MGUS.

^i^ Not tested in 16 CIDP patients with IgG MGUS and 158 CIDP patients without MGUS.

^j^ Not tested in 11 CIDP patients with IgG MGUS and 107 CIDP patients without MGUS.

^k^ Only assessed for patients included in EMC or Amsterdam UMC (IgG MGUS: *n* = 14; no MGUS *n* = 151).

^l^ Based on electrodiagnostic criteria, as described in 2010 EFNS/PNS guidelines for CIDP.

^m^ Based on electrodiagnostic criteria combined with supportive criteria, as described in 2010 EFNS/PNS guidelines for CIDP.

^n^ Defined using 2021 EAN/PNS guideline for CIDP.

^o^ ≥ 1 motor nerve conduction criterium met.

^p^ Motor nerve conduction criteria met in only one nerve.

^q^ Motor nerve conduction criteria not met.

^r^ Based on motor and sensory nerve conduction criteria per clinical subtype, as described in 2021 EAN/PNS guidelines for CIDP.

^s^ Based on motor and sensory nerve conduction criteria combined with supportive criteria, per clinical subtype, as described in 2021 EAN/PNS guidelines for CIDP.

**FIGURE 2:**
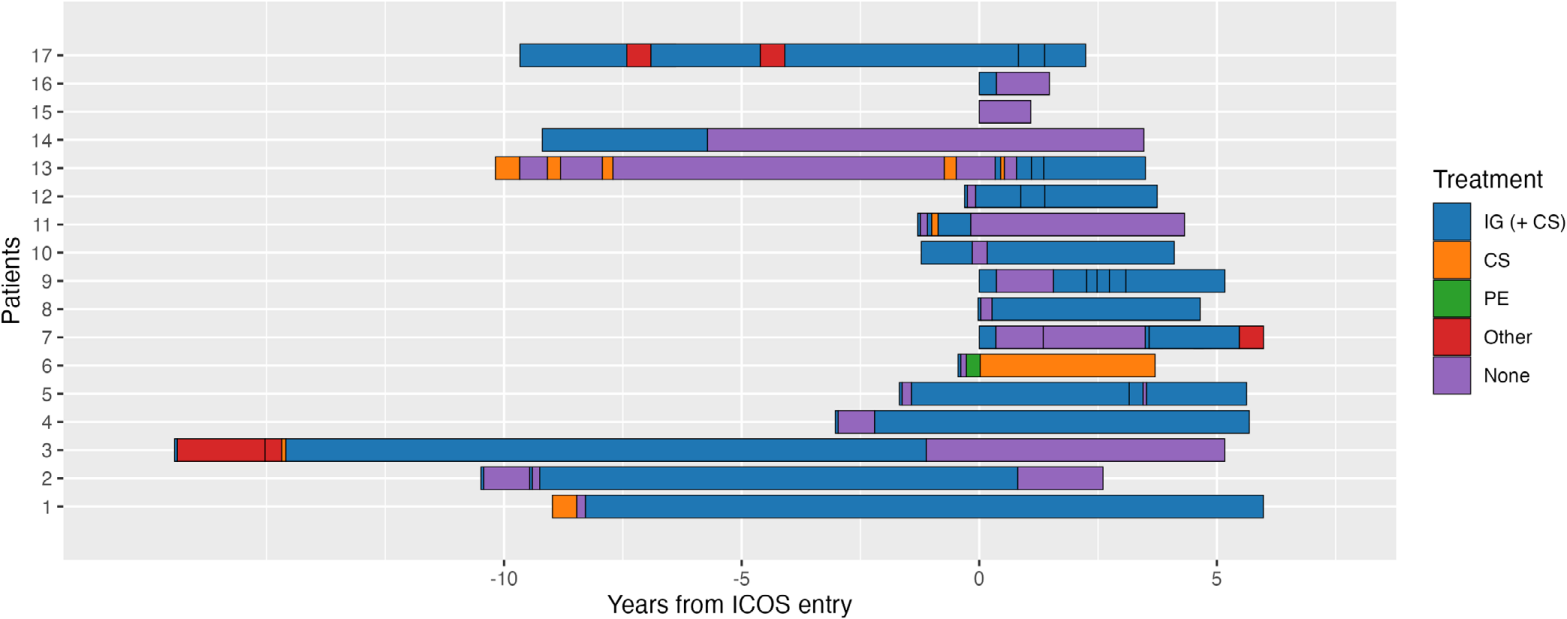
Treatment history of patients with CIDP and IgG MGUS. On the *y*‐axis, the different patients with IgG MGUS are presented. On the *x*‐axis, the time in years is presented, on which the different treatments are indicated by color. The “0” on the *x*‐axis indicates the moment of inclusion into ICOS. In addition: Patient 7 received deep brain stimulation for tremor 16 months after diagnosis; Patient 6 received corticosteroids for kidney transplantation. Specification of “other treatment”: Patient 3: methotrexate, mycophenolate mofetil; Patient 7: prednisolone and methotrexate; Patient 17: PATH trial, fingolimod trial. Abbreviations: CIDP: chronic inflammatory demyelinating polyneuropathy; CS: corticosteroids monotherapy; ICOS: International CIDP Outcome Study; IG (+CS): immunoglobulins with or without corticosteroids; PE: plasmapheresis.

Treatment response rates (per treatment type) of patients with IgG MGUS and patients without MGUS can be found in Table 2. Of patients with IgG MGUS, 80% (12/15) responded to the first treatment compared to 67% (94/140) of patients without MGUS (*p* = 0.39). The treatment history of patients with IgG MGUS is illustrated in Figure 2. Over a median follow‐up of 6 years (IQR: 4–13) from starting first treatment to last ICOS follow up, 56% (9/16) of IgG MGUS patients had at least one period of remission.

### 3.3 Clinical Characteristics, Diagnostic Investigations and Treatment Response in Patients With CIDP and IgM MGUS

All six patients with CIDP and IgM MGUS were male (data not shown). In patients with IgM MGUS, the clinical phenotype was typical in 50% (3/6) compared to 69% (115/165) in patients without MGUS (*p* = 0.20 when comparing all clinical phenotype categories). Atypical phenotypes in patients with IgM MGUS included (multi)focal variants (1/6) and motor variants (2/6). Due to the small sample size, other outcomes were not compared.

Most patients were not assessed for antibodies during their diagnostic work‐up, but most were tested to assess the overall prevalence of paranodal antibodies in our cohort. Paranodal antibodies were tested in 111 patients (58%) and found positive in 8 patients. Paranodal antibodies were predominantly of the IgG4 subclass in all 8/8 patients. None of these patients had IgG MGUS; one patient with IgM MGUS tested positive for anti‐NF155 (Table 3).

### 3.4 Sensitivity Analysis

We selected 153/192 CIDP patients for our sensitivity analyses. We excluded 18 patients enrolled in the UMCU because we did not have access to detailed NCS reports. In addition, eight patients with paranodal antibodies and 15 patients who were classified as “no CIDP” were excluded (one patient both had paranodal antibodies and was classified as “no CIDP”). Results can be found in Tables [Link], [Link], and Figure S1. In general, we found similar results when comparing group differences using these selection criteria. However, the difference in sensory deficits at onset between CIDP patients with IgG MGUS and patients without MGUS did not reach significance in this sensitivity analysis.

## 4 Discussion

We found a higher prevalence of IgG paraproteinemia in patients with CIDP than in matched neurological controls, while the prevalence rates of IgM and IgA paraproteinemia were similar. Patients with CIDP and IgG MGUS differed from patients without MGUS in terms of less frequent acute onset, more frequent sensory deficits at onset, and possibly more frequent distal demyelinating features, indicating a weak association of IgG MGUS with certain CIDP disease features. Treatment response to the first treatment was similar between these groups. These findings indicate that, although IgG MGUS is associated with CIDP, the presence of IgG MGUS does not constitute a distinct subgroup with unique clinical features or treatment implications.

Previous studies, varying in used screening techniques, inclusion criteria, and sample characteristics, reported an IgG paraproteinemia prevalence of 0%–17% in patients with CIDP [7, 21, 22, 23, 24, 25, 26, 27, 28, 29, 30]. In most studies, the prevalence of IgM paraproteinemia seemed higher than IgG paraproteinemia in patients with CIDP, but those studies did not exclude patients with anti‐MAG, unlike ours [24, 26, 27, 28, 31]. No studies have compared the prevalence of each paraprotein isotype separately to a disease control group, but associations between CIDP and MGUS of any isotype have been previously reported, including in a large population study [7, 8]. Our study did not compare MGUS prevalence rate in CIDP to the general population. However, previously reported MGUS prevalence rates in the general population are 3.7% among males aged 60–69 years [6], which appears lower than the prevalence observed in our CIDP sample (median age 57 years, 69% males). A previous study on the clinical characteristics of patients with CIDP and IgG MGUS (and IgA MGUS—these are generally combined in literature) also reported more sensory deficits and low rates of acute disease onsets, and in addition less weakness and disability, compared to idiopathic or diabetic patients with CIDP [32]. Other studies reported no differences except for more frequent cranial nerve involvement in IgG MGUS or IgA MGUS patients compared to patients without MGUS [7]. Previous studies as well as our own found no significant difference in treatment response between patients with CIDP with or without IgG or IgA MGUS [7, 32]. A pooled analysis showed that 80% of all reported IgG MGUS patients with demyelinating polyneuropathy responded to various treatments, similar to our findings [33]. Altogether, these results suggest that first‐line treatments in patients with CIDP seem to be equally effective in patients with IgG MGUS.

Patients with IgG MGUS met the (electro)diagnostic criteria of CIDP as frequently as patients without MGUS. Patients with IgG MGUS more often fulfilled motor criterion “distal CMAP duration prolongation in at least one nerve and at least one other demyelinating parameter in at least one other nerve” compared to patients without MGUS while distal motor latencies were frequently normal in IgG MGUS patients. No previous studies compared NCS results between patients with CIDP with and without IgG MGUS. This finding may indicate more frequent patchy demyelination in the distal nerve segments that affects some but not all motor fibers in CIDP with IgG MGUS patients. Distal nerve segments, like proximal nerve roots, have an impaired nerve blood barrier and it could be speculated that IgG MGUS is a marker for an auto‐antibody mediated pathophysiology targeting epitopes in the distal nerve segment. This is supported by some, but not all biopsy studies [34, 35, 36, 37].

Other possible pathophysiological mechanisms linking MGUS and CIDP, may be related to the known association between MGUS and (the history of) various other auto‐immune disease, such as rheumatoid arthritis [8, 38]. Proposed mechanisms include a shared (genetic or environmental) susceptibility for both diseases, or chronic antigen stimulation by activated immune cells in auto‐immune diseases that triggers development of MGUS [38, 39, 40] Indeed, it has been suggested that MGUS may arise during the CIDP disease course [24, 41, 42]. In our study, the time from onset to diagnosis between patients with or without IgG was similar. We were not able to study the incidence of MGUS during the CIDP disease course as this was not assessed in most patients.

Whether a causal relation between IgM MGUS without (high titers of) anti‐MAG and demyelinating neuropathy exists also remains a matter of debate. Opinions regarding the classification of these patients vary. Authors of the 2021 EAN/PNS guidelines of CIDP state that insufficient evidence supports a difference between CIDP with IgM MGUS without anti‐MAG and CIDP without MGUS [19]. In contrast, a study of 53 patients observed that patients with distal demyelinating neuropathies and IgM MGUS either with or without anti‐MAG, were similar [28]. In our study, the subgroup of patients with CIDP and IgM was too small to allow for meaningful comparisons. Of note, there were no patients with IgM MGUS that had a distal CIDP phenotype that might suggest that these Dutch patients might be more often classified as IgM related neuropathy, regardless of their anti‐MAG status. Alternatively, presence of (high level) of anti‐MAG antibodies without IgM paraprotenemia in some patients with CIDP has recently been debated [43], illustrating the need of better boundaries of diagnosis CIDP and IgM related neuropathy.

Our study has some limitations. First, the control group differed slightly from the patients with CIDP in factors that may influence prevalence rates: screening methods for paraproteins varied, and neurological controls were somewhat older, which might slightly underestimate the true difference in paraprotein prevalence between CIDP patients and controls. Second, the cause for the paraprotein was often not confirmed by BM examinations. Therefore, an underlying malignancy rather than MGUS cannot be excluded. However, BM examinations were likely frequently deferred, as most patients had low paraprotein concentrations, suggesting a low risk of malignancy. Third, both patients with CIDP with an acute clinical presentation as well as those with relapsing–remitting or chronic progressive disease courses may be overrepresented in our sample. This may be explained by our study sites (tertiary centers with expertise in Guillain‐Barré syndrome) and by the ICOS inclusion criteria (i.e., both newly and previously diagnosed patients). However, this did not seem to have influenced the prevalence rate of IgG paraproteinemia, as this was also 9% in the subgroup of newly diagnosed patients with CIDP at study entry. Other limitations include the use of retrospective data in most patients and the limited sample size of patients with IgG MGUS and especially IgM MGUS, which limited drawing firm conclusions regarding phenotype and treatment response in these patients. The sample of treatment naïve patients was also small, which precluded robust analyses of treatment effect in new patients at ICOS entry, using prospectively collected outcome measures. Also, ICOS inclusion criteria were based on the 2010 diagnostic criteria for CIDP [10], which have been updated in 2021 [19]. We applied a sensitivity analysis using a selected group based on the 2021 criteria, which limited our sample size since detailed full NCS reports were not available for all patients. The difference in sensory deficits did not remain significant in this analysis, likely due to the power (as the percentages of patients with sensory deficits were almost identical in the selected and unselected group). Additionally, some diagnostic investigations (e.g., nerve ultrasounds) were only assessed in a small proportion of patients. Finally, in some patients we could not retrieve data on monoclonal testing from referring hospital. These were typically patients with prolonged duration of maintenance treatment. In the recent EAN/PNS 2021 criteria, there is emphasis on testing for monoclonal proteins and vascular endothelial growth factor in the diagnostic work‐up to exclude CIDP mimics, such as POEMS syndrome. Given the long‐term follow‐up in our patients, we are confident that our patients did not have an alternative diagnosis, including those in whom diagnostic data on monoclonal proteins was lacking.

With regard to future directions, larger international database studies using prospective clinical data and biomaterials, such as Inflammatory Neuropathy Consortium Base (INCbase) [44], may help to further investigate and potentially delineate subtypes within the CIDP spectrum sharing immunopathological features using paraprotein, biomarkers, and auto‐antibodies testing in serum and CSF.

In conclusion, IgG MGUS is more prevalent in CIDP than in controls. The presence of IgG MGUS is weakly associated with some CIDP disease features, but response to first‐line treatments is similar in CIDP patients with IgG MGUS compared to those without MGUS. These findings indicate that CIDP is associated with IgG MGUS, and possibly indicate a distinct underlying pathophysiological mechanism in these patients. However, CIDP with IgG MGUS does not constitute a distinct clinical subgroup with unique features or treatment implications. Moreover, MGUS does require evaluation to exclude CIDP mimics and requires careful follow‐up because it bears the risk of malignant transformation.

## Funding

We are grateful to CSL‐Behring, Grifols, and the GBS/CIDP Foundation International for sponsoring ICOS and the current study by unrestricted grants. The sponsors had no influence on the study design, data collection, analysis, conclusions, writing, and submission of the study.

## Conflicts of Interest

M.C. Broers has received a grant from the Dutch Prinses Beatrix Spierfonds for the present manuscript. W.L. van der Pol has received a consulting fee for service to employer from Argenx, outside the submitted work. B.C. Jacobs has received grants from Horizon 2020, Prinses Beatrix Spierfonds, and GBS‐CIDP Foundation International for studies in CIDP outside of the submitted work. He is a member of the board of INCBase. His institution has received funding from Annexon, Hansa Biopharma, Roche, CSL Behring, Grifols, and Octapharma for research in CIDP and/or GBS. He is vice‐chairman of the Medical Advisory Board of the GBS‐CIDP Foundation International. P.A. Van Doorn has received grants or contracts from Dutch Prinses Beatrix Spierfonds and ZonMW, consulting fees from Annexon, Argenx, Hansa Biopharma, Octapharma, Sanofi, and Takeda, payment or honoraria for lectures, presentations, speakers bureaus, manuscript writing or educational events, and support for attending meetings and/or travel from Argenx, Grifols, and Octapharma, and participated on the Data Safety Monitoring Board‐Advisory Board of Annexon, Argenx, Hansa Biopharma, Octapharma, and Sanofi, all outside the submitted work. All grants and fees were paid to his institution. C. Verhamme received research grants from Prinses Beatrix Spierfonds, GBS‐CIDP Foundation International, and the Dutch Research Council (NWO‐OTP) and an honorarium from Neuromuscular ultrasound courses—Dutch society of clinical neurophysiology and Neuromuscular ultrasound courses—Sonoskills, all paid to the institution and outside the submitted work. Also, he is an unpaid board member of the ERN EURO‐NMD and unpaid medical adviser of Spierziekten Nederland—patient organization. J.M.I. Vos has received the following as institutional honoraria: research support from Beigene and AbbVie/Genmab; advisory board/consultancy fees from Sanofi and Janssen; and speaker fees from BMS, Sanofi, Beigene, Novartis, and Amgen. F. Eftimov has received other grants from ZonMw and Prinses Beatrix Spierfonds for studies in CIDP, outside of the submitted work. As principal investigator of INCbase, he also reports investigator‐initiated grants from Kedrion, CSL‐Behring, Grifols, and Takeda Pharmaceutical Company. His institution has received fees from Dianthus, CSL Behring, Grifols, and Takeda for advisory board membership and/or lectures. All grants and fees were paid to his institution. He is a member of the Cochrane Neuromuscular Editorial Board. Authors declare no conflicts of interest.

## Supporting information


**Figure S1:** Motor nerve conduction criteria fulfillment of patients with CIDP with IgG MGUS and without MGUS (sensitivity analysis).

**Table S1:** Details of patients with paraproteinemia.

**Table S2:** Paraprotein screening of patients with CIDP and neurological controls (sensitivity analysis).

**Table S3:** Clinical characteristics and treatment response of patients with CIDP with IgG MGUS and without MGUS (sensitivity analysis).

**Table S4:** Results of diagnostic investigations of patients with CIDP, with IgG MGUS and without MGUS (sensitivity analysis).

## Data Availability

The data that support the findings of this study are available from the corresponding author upon reasonable request.
