# Supplementary figures and images for: CIDP With and Without Monoclonal Gammopathy of Undetermined Significance (MGUS): Comparison of Clinical Phenotype, Diagnostic Features, and Treatment Response
Source: J Peripher Nerv Syst. 2026 Mar 12;31(1):e70116. doi: 10.1111/jns.70116 (PMC12981947; doi:10.1111/jns.70116)

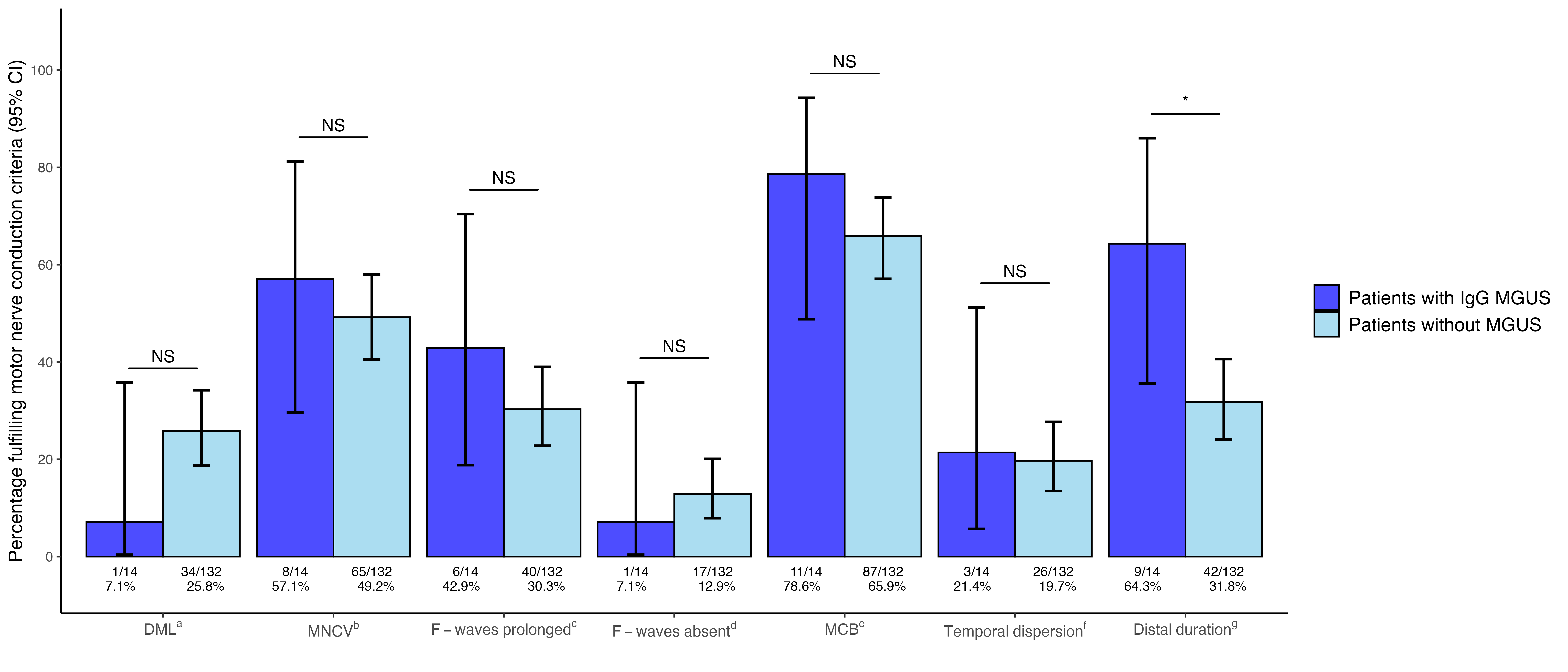

Supplement: Supplementary file 1 — Figure S1: Motor nerve conduction criteria fulfillment of patients with CIDP with IgG MGUS and without MGUS (sensitivity analysis). [file JNS-31-0-s005.png]
