# Supplementary material for: CIDP With and Without Monoclonal Gammopathy of Undetermined Significance (MGUS): Comparison of Clinical Phenotype, Diagnostic Features, and Treatment Response
Source: J Peripher Nerv Syst. 2026 Mar 12;31(1):e70116. doi: 10.1111/jns.70116 (PMC12981947; doi:10.1111/jns.70116)
Supplement: Supplementary file 2 — Table S1: Details of patients with paraproteinemia. [file JNS-31-0-s001.docx]

Supplementary table 1: Details of patients with paraproteinemia

| Patient number^^[[1]](#endnote-1)^^ | Age at screening | Gender | Comorbidity at screening | Diagnosis (phenotype) | Method of screening | Paraprotein isotype | Quantity (g/L) | Bone marrow examination | Hematological diagnosis of paraprotein | Malignant transformation during FU | Time of FU from paraprotein screening (years) | Antibody testing^^[[2]](#endnote-2)^^ | Exclusion for sensitivity analyses (reason) |
| --- | --- | --- | --- | --- | --- | --- | --- | --- | --- | --- | --- | --- | --- |
| 1 | 70 | M | Transient Ischemic Attack | CIDP (typical) | IFX and serum EF | IgG kappa | Paraprotein: <5  IgG: 13.8 (n) | Supportive of MGUS | Confirmed MGUS | No | 14 | Anti-GM1: excluded  Anti-CASPR1: excluded  Anti-CNTN1: excluded  Anti-NF155: excluded | Included |
| 2 | 35 | M |  | CIDP (typical) | IFX and serum EF | IgG kappa | Paraprotein: Not quantifiable  IgG: 9.0 (n) | Not assessed | Unconfirmed MGUS | No | 14 | Anti-GM1: excluded  Anti-GQ1b and anti-GD1b: excluded | Included |
| 3 | 52 | F | Epilepsy | CIDP (typical) | IFX and serum EF | IgG kappa | Paraprotein: < 5  IgG: unknown | Not assessed | Unconfirmed MGUS | No | 21 | Anti-CASPR1: excluded  Anti-CNTN1: excluded  Anti-NF155: excluded | Included |
| 4 | 68 | M | - | CIDP (typical) | EF | IgG kappa | Paraprotein: 4  IgG: 10.2 (n) | Not assessed | Unconfirmed MGUS | No | 8 | Anti-GM1: excluded  Anti-CASPR1: excluded  Anti-CNTN1: excluded  Anti-NF155: excluded | Included |
| 5 | 65 | F | Paroxysmal AF, benign cystadenoma of ovary | CIDP (typical) | IFX and serum EF | IgG lambda | Paraprotein: 3.6 | Not assessed | Unconfirmed MGUS | No | 7 | Anti-GM1: excluded  Anti-CASPR1: excluded  Anti-CNTN1: excluded  Anti-NF155: excluded | Included |
| 6 | 64 | M | Polycystic Kidney Disease | CIDP (typical) | EF | IgG kappa | Paraprotein: Not quantifiable  IgG: 11.9 (n) | Supportive of MGUS | Confirmed MGUS | No^^[[3]](#endnote-3)^^ | 3 | Anti-CASPR1: excluded  Anti-CNTN1: excluded  Anti-NF155: excluded | Included |
| 7 | 67 | M | Colon carcinoma, in remission | CIDP (typical) | IFX and serum EF | IgG lambda | Paraprotein: 5.2 | Assessment in other hospital | Unconfirmed MGUS | No | 5 | Anti-CASPR1: excluded  Anti-CNTN1: excluded  Anti-NF155: excluded | Included |
| 8 | 57 | M | Essential Thrombocythemia, liver cirrhosis in non-cirrhotic portal hypertension due to microthrombi | CIDP (distal) | IFX and serum EF | IgG kappa | Paraprotein: Not quantifiable  IgG: 19.5 (h) | Not assessed | Unconfirmed MGUS | No | 4 | Anti-CASPR1: excluded  Anti-CNTN1: excluded  Anti-NF155: excluded | Included |
| 9 | 61 | M | - | CIDP (typical) | IFX and serum EF | IgG kappa | Paraprotein: 1 | Not assessed | Unconfirmed MGUS | No | 4 | Anti-CASPR1: excluded  Anti-CNTN1: excluded  Anti-NF155: excluded | Included |
| 10 | 73 | F | Hyperthyroidism, colon carcinoma in remission,  type 2 diabetes mellitus | CIDP (typical) | IFX and serum EF | IgG lambda | Paraprotein: Not quantifiable  IgG: not assessed | Not assessed | Unconfirmed MGUS | No | 5 | Anti-GM1: excluded  Anti-GQ1b and anti-GD1b: excluded | Excluded (No NCS available for UMCU patients) |
| 11 | 60 | M | Degenerative back complaints | CIDP ((multi) focal) | IFX and serum EF | IgG lambda | Paraprotein: Not quantifiable  IgG: not assessed | Supportive of MGUS | Confirmed MGUS | No | 5 | Anti-GM1: demonstrated  Anti-GQ1b: excluded | Excluded (No NCS available for UMCU patients) |
| 12 | 46 | F | - | CIDP (typical) | Protein spectrum | IgG lambda | Paraprotein: 4 | Not assessed or assessed in other hospital | Unconfirmed MGUS | No | 3 | Anti-CASPR1: excluded  Anti-CNTN1: excluded  Anti-NF155: excluded | Included |
| 13 | 69 | M | - | CIDP (typical) | EF | IgG kappa | Paraprotein: Not quantifiable  IgG: 9.4 (n) | Not assessed | Unconfirmed MGUS | Multiple myeloma^^[[4]](#endnote-4)^^ | 3 |  | Included |
| 14 | 49 | F | - | CIDP (typical) | Unknown | IgG lambda | Paraprotein: 3 | Not assessed or assessed in other hospital | Unconfirmed MGUS | No | 14 |  | Excluded (No NCS available for UMCU patients) |
| 15 | 71 | M | - | CIDP (typical) | Capillary EF | IgG lambda | Paraprotein: 2 | Not assessed | Unconfirmed MGUS | No | 2 |  | Included |
| 16 | 62 | M | - | CIDP ((predominantly) motor) | IFX and serum EF | IgG lambda | Paraprotein: Not quantifiable  IgG: 7.8 (n) | Supportive of MGUS | Confirmed MGUS | No | 1 |  | Included |
| 17 | 48 | M | - | CIDP (typical) | IFX and serum EF | IgG kappa | Paraprotein: 2 | Not assessed | Unconfirmed MGUS | No | 11 |  | Included |
| 18 | 58 | M | Hiv infection | CIDP (typical) | IFX and serum EF | IgM kappa | Paraprotein: Not quantifiable  IgM: 1.14 (n) | Supportive of MGUS | Confirmed MGUS | No | 6 | Anti-GM1: excluded  Anti-CASPR1: excluded  Anti-CNTN1: excluded  Anti-NF155: excluded | Excluded (No CIDP based on diagnostic 2021 criteria) |
| 19 | 41 | M | Vitamin B12 deficiency | CIDP ((multi) focal) | IFX and serum EF | IgM lambda | Paraprotein: Not quantifiable  IgM: 3.0 (h) | Supportive of MGUS | Confirmed MGUS | No | 10 | Anti-CASPR1: excluded  Anti-CNTN1: excluded  Anti-NF155: excluded | Included |
| 20 | 75 | M | Guillain-Barré Syndrome**,**  Crohn’s disease, nephrolithiasis, renal insufficiency | CIDP (typical) | Capillary EF | IgM kappa | Paraprotein: Not quantifiable  IgM: not assessed  Total gamma: 10 (n) | Supportive of MGUS | Confirmed MGUS | No^^[[5]](#endnote-5)^^ | 1 | Anti-CASPR1: excluded  Anti-CNTN1: excluded  Anti-NF155: excluded | Included |
| 21 | 59 | M | - | CIDP (motor) | EF | IgM kappa | Paraprotein: Not quantifiable  IgM 1.15 (n) | Not assessed | Unconfirmed MGUS | No^^[[6]](#endnote-6)^^ | 15 | Anti-CASPR1: excluded  Anti-CNTN1: excluded  Anti-NF155: excluded | Excluded (No CIDP based on diagnostic 2021 criteria) |
| 22 | 69 | M | - | CIDP (typical) | Unknown | IgM kappa | Paraprotein: 2 | Assessment in other hospital | Waldenström's Macroglobulinemia | - | 5 | Anti-CASPR1: excluded  Anti-CNTN1: excluded  Anti-NF155: excluded | - |
| 23 | 51 | M | Renal cell carcinoma treated with nefrectomy | CIDP (typical) | Capillary EF | IgM lambda | Paraprotein: Not quantifiable  IgM: 0.83 (n) | Supportive of MGUS | Confirmed MGUS | No | 3 | Anti-CASPR1: excluded  Anti-CNTN1: excluded  Anti-NF155: excluded | Included |
| 24 | 24 | M | - | CIDP (motor) | Unknown | IgM kappa | Paraprotein: Not quantifiable  IgM: unknown | Not assessed or assessed in other hospital | Unconfirmed MGUS | No | 4 | Anti-GM1: excluded  Anti-GQ1b and anti-GD1b: excluded  Anti-CASPR1: excluded  Anti-CNTN1: excluded  Anti-NF155: demonstrated | Excluded (NF155 antibodies) |
| 25 | 67 | M | - | CIDP (typical) | Unknown | Biclonal: IgG lambda and  IgM lambda | Paraprotein: 2.9  Paraprotein: 1.9 | Supportive of MGUS | Confirmed MGUS | No | 5 | Anti-GM1: excluded  Anti-CASPR1: excluded  Anti-CNTN1: excluded  Anti-NF155: excluded | Included |
| 26 | 75 | M | Type 2 diabetes, arthritis of the foot, embolism in the hand, left bundle branch block | CIDP (typical) | IFX and serum EF | Free light chains kappa | Free light chains kappa: 42.4 (h)  Free kappa/lambda ratio: 1.92 (h) | Not assessed | Unconfirmed MGUS^^[[7]](#endnote-7)^^ | No | 5 |  | Excluded (No NCS available for UMCU patients) |
| Patient number | Age at screening | Gender | Comorbidity at screening | Diagnosis | Method of screening | Paraprotein isotype | Quantity (g/L) | Bone marrow examination | Hematological diagnosis of paraprotein | Malignant transformation during FU | Time of FU from paraprotein screening (years) | Antibody testing^2^ | Exclusion for sensitivity analyses (reason) |
| 27 | 75 | F | NA | AN | IFX and serum EF | IgG lambda | Paraprotein: 1.3 | Not assessed or assessed in other hospital | Unconfirmed MGUS | NA | NA | NA | NA |
| 28 | 57 | M | NA | MND | IFX and serum EF | IgG lambda | Paraprotein: 8 | Not assessed or assessed in other hospital | Unconfirmed MGUS | NA | NA | NA | NA |
| 29 | 68 | M | NA | AN | IFX and serum EF | IgG lambda | Paraprotein: 12 | Supportive of MGUS | Confirmed MGUS | NA | NA | NA | NA |
| 30 | 66 | M | NA | AN | IFX and serum EF | IgG kappa | Paraprotein: 3.9 | Not assessed | Unconfirmed MGUS | NA | NA | NA | NA |
| 31 | 73 | F | NA | AN | EF | IgG kappa | Paraprotein: Unknown | Not assessed | Unconfirmed MGUS | NA | NA | NA | NA |
| 32 | 57 | M | NA | AN | IFX and serum EF | IgG kappa | Paraprotein: 4 | Supportive of MGUS | Confirmed MGUS | NA | NA | NA | NA |
| 33 | 80 | M | NA | MND | IFX and serum EF | IgM lambda | Paraprotein: 1.7 | Supportive of MGUS | Confirmed MGUS | NA | NA | NA | NA |
| 34 | 80 | M | NA | MND | IFX and serum EF | IgM lambda | Paraprotein: 10.5  IgM: 11.06 (h) | Supportive of MGUS | Confirmed MGUS | NA | NA | NA | NA |
| 35 | 71 | M | NA | MND | IFX and serum EF | IgM kappa | Paraprotein: 1.1 | Supportive of MGUS | Confirmed MGUS | NA | NA | NA | NA |
| 36 | 45 | F | NA | MND | IFX and serum EF | IgA lambda | Paraprotein: < 0.1 | Not assessed | Unconfirmed MGUS | NA | NA | NA | NA |
| 37 | 72 | M | NA | MND | IFX and serum EF | IgA lambda | Paraprotein: 0.8 | Not assessed | Unconfirmed MGUS | NA | NA | NA | NA |
| 38 | 62 | M | NA | AN | IFX and serum EF | Biclonal: IgG lambda and  IgA lambda | Paraprotein (IgG): 2.1  IgG: 13.5 (n)  Paraprotein (IgA): Not quantifiable  IgA: 3.91 (n) | Supportive of MGUS | Confirmed MGUS | NA | NA | NA | NA |
| 39 | 73 | M | NA | MND | IFX and serum EF | Biclonal: IgM kappa and  IgM lambda | Paraprotein (IgM kappa): 0.4  Paraprotein (IgM lambda): 0.2 | Supportive of MGUS | Confirmed MGUS | NA | NA | NA | NA |

Abbreviations: AF: atrial fibrillation; AI: autoimmune; AN: axonal neuropathy; CIDP: chronic inflammatory demyelinating polyneuropathy; EF: electrophoresis; F: female; FU: follow-up; h: high (above normal range); IFX: immunofixation; Ig: immunoglobulin; κ: kappa; λ: lambda; MND: motor neuron disease; M: male; MGUS: monoclonal gammopathy of undetermined significance; NA: not applicable; NCS: nerve conduction studies; n: normal (within reference range); UMCU: University Medical Center Utrecht.

1. Patient numbers were **anonymized;** [↑](#endnote-ref-1)
2. Results of testing of anti-GM1, anti-GQ1b, anti-GD1b, anti-CASPR1, anti-CNTN1, and anti-NF155 are presented, only when assessed; [↑](#endnote-ref-2)
3. Patient 6 developed Epstein-Barr Virus related Post-Transplant Lymphoproliferative Disorder (PTLD) with central nervous system localization, resulting from immunosuppression with tacrolimus, Mycophenolate Mofetil and prednisone. This was regarded to be unrelated to MGUS. [↑](#endnote-ref-3)
4. Multiple myeloma diagnosed 3 years after screening; [↑](#endnote-ref-4)
5. Patient 20 had DAT-positive hemolytic anemia due to cold agglutinins, possibly based on monoclonal B-cell lymphocytosis, managed with watchful waiting. Patient died of complications of Morbus Crohn; [↑](#endnote-ref-5)
6. Patient 21 was diagnosed with myelodysplastic syndrome 15 years after paraprotein screening, which was regarded as unrelated to the paraprotein; [↑](#endnote-ref-6)
7. Patient 26 was suspected of a lymphoma (retroperitoneal), pathological examination showed an extragonadal seminoma. [↑](#endnote-ref-7)
