# Supplementary material for: CIDP With and Without Monoclonal Gammopathy of Undetermined Significance (MGUS): Comparison of Clinical Phenotype, Diagnostic Features, and Treatment Response
Source: J Peripher Nerv Syst. 2026 Mar 12;31(1):e70116. doi: 10.1111/jns.70116 (PMC12981947; doi:10.1111/jns.70116)
Supplement: Supplementary file 3 — Table S2: Paraprotein screening of patients with CIDP and neurological controls (sensitivity analysis). [file JNS-31-0-s002.docx]

Supplementary table 2: Paraprotein screening of patients with CIDP and neurological controls (sensitivity analysis)

|  | CIDP (n=153) | Neurological controls (n=192)^^[[1]](#footnote-1)^^ | *p*-value |
| --- | --- | --- | --- |
| Age at screening  Gender (males) | 59 years (13)  69% (106/153) | 63 years (13)  64% (122/192) | **0.01**  0.30 |
| Method of screening^^[[2]](#footnote-2)^^ |  |  |  |
| Serum IFX and EF  Pentascreening  Capillary EF  EF or protein spectrum | 49% (67/137)  1% (1/137)  11% (15/137)  39% (54/137) | 69% (127/184)  1% (1/184)  1% (1/184)  30% (56/184) | **<0.01**^^[[3]](#footnote-3)^^ |
| Prevalence |  |  |  |
| **IgG**   - LC type   **IgM**   - LC type   **IgA**   - LC type   **Biclonal**   - - IgG λ and IgM λ - - IgG λ and IgA λ - - IgM λ and IgM κ   **Free light chains** | 9% (5 - 14%; 14/153)   - 64% κ; 36% λ   3% (0 – 5%; 4/153)   - 50% κ; 50% λ   0% (0 – 0%; 0/153)  1% (0 – 2%; 1/153)^^[[4]](#footnote-4)^^   - 100% (1/1) - 0% (0/1) - 0% (0/1)   0% (0 – 0%; 0/153) | 3% (1 – 6%; 6/192)   - 50% κ; 50% λ   2% (0 – 3%; 3/192)^^[[5]](#footnote-5)^^   - 67% κ; 33% λ   1% (0 – 2%; 2/192)   - 100% κ   1% (0 – 2%; 2/192)^^[[6]](#footnote-6)^^   - 0% (0/2) - 50%(1/2) - 50% (1/2)   0% (0-0%; 0/192)^^[[7]](#footnote-7)^^ | **0.02**  0.70  0.51  0.51  1.00 |
| Paraprotein concentration^^[[8]](#footnote-8)^^ |  |  |  |
| Not quantifiable  <5 g/L  5 - 10 g/L  10 - 15 g/L  >15 g/L | 42% (8/19)  53% (10/19)  5% (1/19)  0% (0/19)  0% (0/19) | 0% (0/12)  75% (9/12)  8% (1/12)  17% (2/12)  0% (0/12) | 0.73^^[[9]](#footnote-9)^^ |
| Hematological diagnosis^^[[10]](#footnote-10)^^ |  |  |  |
| MGUS   - Confirmed - Unconfirmed   B-cell or plasma cell malignancy | 95% (18/19)   - 39% (7/18) - 61% (11/18)   5% (1/19)^^[[11]](#footnote-11)^^ | 100% (13/13)   - 54% (7/13) - 46% (6/13)   0% (0/13) | 1.00 |
| Malignant transformation during FU | 6% (1/18)^^[[12]](#footnote-12)^^ | NA | NA |

Numeric data are presented as means and standard deviations (for normal distributed data; mean (SD)) or medians and interquartile range (for non-normally distributed data; median (interquartile range)). Categorical variables are presented as percentages (with 95% confidence intervals for paraprotein prevalence rates) and count/total (percentage (95% CI; count/total) or percentage (count/total)).

Abbreviations: CIDP: chronic inflammatory demyelinating polyneuropathy; EF: electrophoresis; FU: follow up; IFX: serum immunofixation; LC: light chain; κ: kappa; λ: lamba.

1. Axonal neuropathy: 38%; motor neuron disease: 62%; [↑](#footnote-ref-1)
2. Method of screening was unknown in 16 ICOS patients and 8 controls; [↑](#footnote-ref-2)
3. With post-hoc pairwise Fisher’s test: significant difference in ‘serum IFX and EF’ and ‘capillary EF’ (p<0.01); [↑](#footnote-ref-3)
4. One patient with both IgM lambda (1.9 g/L) and IgG lambda (2.9 g/L) paraproteins; [↑](#footnote-ref-4)
5. One patient with an IgM lambda paraprotein had also dubious IgG lambda (g/L not quantifiable) paraprotein. In addition: one patient had a dubious IgM kappa paraprotein (g/L not quantifiable) and one patient had a dubious IgM lambda paraprotein (g/L not quantifiable); [↑](#footnote-ref-5)
6. One patient with both IgG lambda (2.1 g/L) and IgA lambda (g/L not quantifiable) paraproteins and one patient with IgM lambda (0.2 g/L) and IgM kappa (0.4 g/L) paraproteins; [↑](#footnote-ref-6)
7. In addition: one patient had dubious elevated free light chain, of which the type light chain was unable to determine (kappa/lambda ratio: 0.49); [↑](#footnote-ref-7)
8. Only for patients with paraproteins. One patient with elevated kappa light chain had a concentration of 42.4 g/L. For patients with biclonal paraproteinemia, we used the largest quantity of the two paraproteins for categorization. Concentration was unknown for one control patient; [↑](#footnote-ref-8)
9. Comparison between categories: **‘<5 g/L’, ‘5-10 g/L’, and ’10-15 g/L’ and ‘>15 g/L’;** [↑](#footnote-ref-9)
10. Hematological diagnosis of paraprotein or elevated free light chains, at around screening, in case a paraprotein or elevated free light chains was found. When paraprotein concentrations were <30 g/L and bone marrow infiltration was <10 %, patients were classified as confirmed MGUS. When paraprotein concentrations were <30 g/L, but no bone marrow examination was executed or available, we classified patients as unconfirmed MGUS; [↑](#footnote-ref-10)
11. One patient with CIDP and IgM paraproteinemia at screening was diagnosed with Waldenström macroglobulinemia within 2 months after screening; [↑](#footnote-ref-11)
12. One patient diagnosed with multiple myeloma diagnosed 3 years after screening. [↑](#footnote-ref-12)
