# Supplementary material for: CIDP With and Without Monoclonal Gammopathy of Undetermined Significance (MGUS): Comparison of Clinical Phenotype, Diagnostic Features, and Treatment Response
Source: J Peripher Nerv Syst. 2026 Mar 12;31(1):e70116. doi: 10.1111/jns.70116 (PMC12981947; doi:10.1111/jns.70116)
Supplement: Supplementary file 4 — Table S3: Clinical characteristics and treatment response of patients with CIDP with IgG MGUS and without MGUS (sensitivity analysis). [file JNS-31-0-s003.docx]

Supplementary table 3: Clinical characteristics and treatment response of patients with CIDP with IgG MGUS and without MGUS (sensitivity analysis)

|  | CIDP with IgG MGUS  *n*=14 | CIDP without  MGUS  *n*=132 | *p*-value |
| --- | --- | --- | --- |
| Gender (males)  Age at CIDP diagnosis  Other AI disease  Onset to ICOS entry  Diagnosis to ICOS entry | 79 % (11/14)  59 years (10)  0% (0/14)  4 years (1 – 10)  1 years (0 – 9) | 68% (90/132)  56 years (16)  16% (21/131)^^[[1]](#footnote-1)^^  2 years (1 – 7)^^[[2]](#footnote-2)^^  1 years (0 – 3) | 0.55  0.33  0.22  0.19  0.49 |
| Phenotype |  |  |  |
| Typical  Variants   - (Multi) focal - Distal - (Predominantly) motor - (Predominantly) sensory | 76% (12/14)  0% (0/14)  7% (1/14)  7% (1/14)  0% (0/14) | 79% (104/132)  12% (16/132)  2% (3/132)  4% (5/132)  3% (4/132) | 0.31 |
| Disease onset |  |  |  |
| Acute clinical presentation  Onset to diagnosis  Weakness  Sensory deficits  Cranial nerve deficits  Gait disturbances | 0% (0/14)  1 year (1 – 1)  29% (4/14)  93% (13/14)  0% (0/14)  21% (3/14) | 36% (48/132)  1 year (0 – 2)  56% (74/132)  67% (88/132)  6% (8/132)  19% (25/132) | **<0.01**  0.13  0.09  0.06  1.00  0.73 |
| Examination at diagnosis |  |  |  |
| Weakness^^[[3]](#footnote-3)^^   - Proximal - Distal   Sensory dysfunction  Ataxia  Tremor  Autonomic dysfunction  Pain | 79% (11/14)  46% (6/13)  77% (10/13)  100% (14/14)  14% (2/14)  14% (2/14)  0% (0/14)  14% (2/14) | 94% (119/127)  69% (87/127)  91% (116/127)  91% (115/126)^^[[4]](#footnote-4)^^  3% (4/132)  15% (20/132)  6% (8/132)  27% (35/132) | 0.08  0.13  0.12  0.60  0.10  1.00  1.00  0.35 |
| Newly diagnosed patients at entry^^[[5]](#footnote-5)^^ | *n*=5 | *n*=44 |  |
| Typical phenotype  MRC-SS (points)^^[[6]](#footnote-6)^^   - Proximal^^[[7]](#footnote-7)^^ - Distal^^[[8]](#footnote-8)^^   Grip strength (kPa)^^[[9]](#footnote-9)^^   - Weakest hand - Strongest hand   INCAT-SS (points)^^[[10]](#footnote-10)^^  I-RODS (centile)^^[[11]](#footnote-11)^^ | 60% (3/5) ^^[[12]](#footnote-12)^^  56 (56 – 60)  38 (38 - 40)  18 (18 - 20)  71 (50 - 78)  86 (60 - 88)  7 (2 - 7)  71 (57 - 83) | 77% (34/44)  54 (50 – 56)  36 (34 – 40)  17 (16 – 18)  45 (30 – 66)  50 (39 – 73)  6 (4 – 8)  61 (49 – 71) | 0.58  0.14  0.29  0.29  0.18  0.18  0.48  0.39 |
| Treatment response |  |  |  |
| Treatment received  Responder^^[[13]](#footnote-13)^^   - IVIg monotherapy - Corticosteriods monotherapy - IVIg and corticosteroids - Other | 93% (13/14)  75% (9/12)   - 71% (5/7) - 100% (2/2) - 67% (2/3)^^[[14]](#footnote-14)^^ - 0/0 | 95% (126/132)  68% (77/114)   - 71% (47/66)^^[[15]](#footnote-15)^^ - 63% (12/19) - 59% (16/27)^^[[16]](#footnote-16)^^ - 100% (2/2)^^[[17]](#footnote-17)^^ | 0.75 |

Numeric data are presented as means and standard deviations (for normal distributed data; mean (SD)) or medians and interquartile range (for non-normally distributed data; median (interquartile range)). Categorical variables are presented as percentages and count/total (percentage (count/total)).

Abbreviations: AI: auto-immune; CIDP: chronic inflammatory demyelinating polyneuropathy; INCAT-SS: modified inflammatory neuropathy cause and treatment sensory score; I-RODS: the inflammatory Rasch-Overall Disability Scale; IVIg: intravenous immunoglobulins; kPa: kilopascal; MRC-SS: Medical Research Council sum score.

1. Unknown for one patient; [↑](#footnote-ref-1)
2. Year of onset unknown in 9 patients. [↑](#footnote-ref-2)
3. Location of weakness unknown for one patient with IgG MGUS and weakness unknown for 5 patients without MGUS; [↑](#footnote-ref-3)
4. Unknown for six patients; [↑](#footnote-ref-4)
5. Newly diagnosed patients at ICOS entry (IgG MGUS: n=5; no MGUS: n=44); [↑](#footnote-ref-5)
6. Ranging from 0 [severe weakness] to 60 points [normal strength]); [↑](#footnote-ref-6)
7. MRC sum score of arm abduction, elbow flexion, hip flexion and knee extension, bilaterally (ranging from 0 [severe weakness] to 40 points [normal strength]); [↑](#footnote-ref-7)
8. MRC sum score of wrist extension and ankle dorsiflexion, bilaterally (ranging from 0 [severe weakness] to 20 points [normal strength]); [↑](#footnote-ref-8)
9. Ranging from 0 [severe weakness] to 160 kPa [no weakness], best out of three measurements; [↑](#footnote-ref-9)
10. Ranging from 0 [no sensory deficits] to 33 points [severe sensory deficits]; [↑](#footnote-ref-10)
11. Ranging from 0 [severe disability] to 100 centiles [no disability]; [↑](#footnote-ref-11)
12. Atypical variants: 1/5 distal, 1/5 pure motor; [↑](#footnote-ref-12)
13. A responder was defined as a patient that improved ≥ 1 point on the mRS upon the first treatment. Based on the available data we were able to classify treatment response in 91 % of patients using the mRS (in 12/13 patients with IgG MGUS and 114/126 of patients without MGUS); [↑](#footnote-ref-13)
14. One patient participated in the OPTIC trail; [↑](#footnote-ref-14)
15. Six patients participated in the OPTIC trial; [↑](#footnote-ref-15)
16. Seven participated in the OPTIC trial; [↑](#footnote-ref-16)
17. One patient was treated with pulsed corticosteroids and plasmapheresis, and one patient was treated with plasmapheresis. [↑](#footnote-ref-17)
