# Supplementary material for: CIDP With and Without Monoclonal Gammopathy of Undetermined Significance (MGUS): Comparison of Clinical Phenotype, Diagnostic Features, and Treatment Response
Source: J Peripher Nerv Syst. 2026 Mar 12;31(1):e70116. doi: 10.1111/jns.70116 (PMC12981947; doi:10.1111/jns.70116)
Supplement: Supplementary file 5 — Table S4: Results of diagnostic investigations of patients with CIDP, with IgG MGUS and without MGUS (sensitivity analysis). [file JNS-31-0-s004.docx]

Supplementary table 4: Results of diagnostic investigations of patients with CIDP, with IgG MGUS and without MGUS (sensitivity analysis)

|  | CIDP with IgG MGUS  *n*=14 | CIDP without  MGUS  *n*=132 | *p*-value |
| --- | --- | --- | --- |
| Serum antibodies |  |  |  |
| Anti-GM1^^[[1]](#footnote-1)^^  Anti-GQ1b or anti-GD1b^^[[2]](#footnote-2)^^ | 0% (0/3)  0% (0/1) | 8% (3/39)  0% (0/18) | NA^^[[3]](#footnote-3)^^ |
| CIDP diagnosis supported by |  |  |  |
| CSF examination^^[[4]](#footnote-4)^^  Nerve ultrasound^^[[5]](#footnote-5)^^  Nerve biopsy^^[[6]](#footnote-6)^^  MRI scan^^[[7]](#footnote-7)^^ | 75% (9/12)  50% (2/4)  0% (0/1)  0% (0/5) | 71% (74/104)  74% (35/47)  50% (2/4)  16% (7/44) | 1.00  0.30  1.00  0.32 |
| NCS |  |  |  |
| Motor nerves tested | 5 (4 – 9) | 6 (4 – 12) | 0.49 |
| Classification 2010 crit |  |  |  |
| Electrodiagnostic crit.^^[[8]](#footnote-8)^^   - Definite CIDP - Probable CIDP - Possible CIDP - No CIDP | 86 % (12/14)  0% (0/14)  14% (2/14)  0% (0/14) | 80% (105/132)  2% (3/132)  11% (15/132)  7% (9/132) | 0.73 |
| Diagnostic crit.^^[[9]](#footnote-9)^^   - Definite CIDP - Probable CIDP - Possible CIDP - No CIDP | 100% (14/14)  0% (0/14)  0% (0/14)  0% (0/14) | 86% (114/132)  5% (6/132)  3% (3/132)  7% (9/132) | 0.16 |
| Classification 2021 crit.^a^ |  |  |  |
| Motor nerve conduction crit.^^[[10]](#footnote-10)^^   - Strongly supportive of dem.^^[[11]](#footnote-11)^^ - Weakly supportive of dem.^^[[12]](#footnote-12)^^ - Not supportive of dem.^^[[13]](#footnote-13)^^ | 93% (13/14)  7% (1/14)  0% (0/14) | 80 % (105/132)  5 % (7/132)  5 % (7/132) | 1.00 |
| Electrodiagnostic crit.^^[[14]](#footnote-14)^^   - CIDP - Possible CIDP - No CIDP | 86% (12/14)  14% (2/14)  0% (0/14) | 72% (95/132)  20% (27/132)  8% (10/132) | 0.70 |
| Diagnostic crit.^^[[15]](#footnote-15)^^   - CIDP - Possible CIDP - No CIDP | 86% (12/14)  14% (2/14)  0% (0/14) | 81% (107/132)  19% (25/132)  0% (0/132) | 1.00 |

Numeric data are presented as means and standard deviations (for normal distributed data; mean (SD)) or medians and interquartile range (for non-normally distributed data; median (interquartile range)). Categorical variables are presented as percentages and count/total (percentage (count/total)).

Abbreviations: CIDP: chronic inflammatory demyelinating polyneuropathy; Crit.: criteria; dem.: demyelination; CSF: cerebrospinal fluid; NCS: nerve conduction studies; MRI: magnetic resonance imaging.

1. Not tested in 11 CIDP patients with IgG MGUS and 93 CIDP patients without MGUS; [↑](#footnote-ref-1)
2. Not tested in 13 CIDP patients with IgG MGUS and 114 CIDP patients without MGUS; [↑](#footnote-ref-2)
3. Differences between groups not assessed for significance due to the small sample size; [↑](#footnote-ref-3)
4. Not tested in 2 CIDP patients with IgG MGUS and 28 CIDP patients without MGUS; [↑](#footnote-ref-4)
5. Not tested in 10 CIDP patients with IgG MGUS and 85 CIDP patients without MGUS; [↑](#footnote-ref-5)
6. Not tested in 13 CIDP patients with IgG MGUS and 128 CIDP patients without MGUS; [↑](#footnote-ref-6)
7. Not tested in 9 CIDP patients with IgG MGUS and 88 CIDP patients without MGUS; [↑](#footnote-ref-7)
8. Based on electrodiagnostic criteria, as described in 2010 EFNS/PNS guidelines for CIDP; [↑](#footnote-ref-8)
9. Based on electrodiagnostic criteria combined with supportive criteria, as described in 2010 EFNS/PNS guidelines for CIDP; [↑](#footnote-ref-9)
10. Defined using 2021 EAN/PNS guideline for CIDP; [↑](#footnote-ref-10)
11. ≥ 1 motor nerve conduction criterium met; [↑](#footnote-ref-11)
12. Motor nerve conduction criteria met in only 1 nerve; [↑](#footnote-ref-12)
13. Motor nerve conduction criteria not met; [↑](#footnote-ref-13)
14. Based on motor and sensory nerve conduction criteria per clinical subtype, as described in 2021 EAN/PNS guidelines for CIDP; [↑](#footnote-ref-14)
15. Based on motor and sensory nerve conduction criteria combined with supportive criteria, per clinical subtype, as described in 2021 EAN/PNS guidelines for CIDP. [↑](#footnote-ref-15)
